# Dietary intake of low-income adults in South Africa: ultra-processed food consumption a cause for concern

**DOI:** 10.1017/S1368980023002811

**Published:** 2024-01-11

**Authors:** Tamryn Frank, Shu Wen Ng, Caitlin M Lowery, Anne-Marie Thow, Elizabeth C Swart

**Affiliations:** 1 School of Public Health, Faculty of Community and Health Sciences, University of the Western Cape, Private Bag X17, Bellville, Cape Town 7535, South Africa; 2 Department of Nutrition, Gillings School of Global Public Health and the Carolina Population Center, The University of North Carolina Chapel Hill, Chapel Hill, USA; 3 Menzies Centre for Health Policy, School of Public Health, Charles Perkins Centre, The University of Sydney, Sydney, NSW, Australia; 4 Department of Dietetics and Nutrition, University of the Western Cape, Cape Town, South Africa; 5 DSI/NRF Centre of Excellence in Food Security, University of the Western Cape, Cape Town, South Africa

**Keywords:** Ultra-processed, South Africa, Dietary quality, Low income, Nutrition policy

## Abstract

**Objective:**

Given the rapidly changing food environment and proliferation of ultra-processed foods (UPF) in South Africa (SA), this study aimed to critically evaluate dietary quality and adequacy of low-income adults using the Nova classification system and WHO and World Cancer Research Fund dietary guidelines.

**Design:**

Secondary household data and 1-d 24-h recalls were analysed from two cross-sectional studies conducted in 2017–2018. Foods consumed were classified according to the Nova classification system. Compliance with WHO dietary guidelines and UPF consumption trends were evaluated.

**Setting:**

Three low-income areas (Langa, Khayalitsha and Mount Frere) in SA were included.

**Participants:**

In total, 2521 participants (18–50 years) were included in the study.

**Results:**

Participants had a mean energy intake of 7762 kJ/d. Most participants were within the acceptable WHO guideline range for saturated fat (80·4 %), total fat (68·1 %), Na (72·7 %) and free sugar (57·3 %). UPF comprised 39·4 % of diets among the average adult participant. Only 7·0 % of all participants met the WHO guideline for fruit and vegetables and 18·8 % met the guideline for fibre. Those within the highest quartile of share of energy from UPF consumed statistically higher amounts of dietary components to limit and were the highest energy consumers overall.

**Conclusions:**

Low-income adults living in SA are consuming insufficient protective dietary components, while UPF consumption is prevalent. Higher UPF consumers consume larger amounts of nutrients linked to increased chronic disease risk. Policy measures are urgently needed in SA to protect against the proliferation of harmful UPF and to promote and enable consumption of whole and less UPF.

The manner in which food is produced, distributed and marketed has changed drastically in recent history. Although food security has improved (prior to the onset of the COVID-19 pandemic), economic development has displaced traditional dietary patterns and driven a shift in food preferences, resulting in the nutrition transition^(1)^. This changing food environment, synonymous with a proliferation of packaged foods high in sugar, salt and saturated fat, otherwise known as ultra-processed foods (UPF), undermines dietary patterns based on minimally and unprocessed food and processed culinary ingredients^(2)^. UPF refer to ‘*formulations mostly of cheap industrial sources of dietary energy and nutrients plus additives, using a series of processes*’^(3)^. These are typically industrially processed foods, high in nutrients known to negatively affect health (Na, saturated and trans-fats and added sugars) and are energy dense^(4)^. These high-energy, low nutritional quality foodstuffs are usually made from cheap ingredients and contain additives such as artificial colourants and flavourants, but are very palatable, require little preparation and are convenient for consumers^(5)^. The entry of large transnational food corporations in the Global South over the last few decades has resulted in rapidly increasing consumption rates of UPF in low- and middle-income countries^(6)^. Consumption habits and choices are continually shifting towards unhealthy UPF due to the price, taste, convenience, availability and marketing strategies employed by large corporations^(2)^.

Although South Africa is classified as an upper-middle income country, it has one of the highest levels of income inequality in the world, with 55 % of the population living in poverty^(7)^, and a continually rising unemployment rate, at 34 % in 2022^(8)^. Given the country’s historical discriminatory past of apartheid, with black people segregated to reside in under-resourced townships with poor access to education and employment, those living in low-income townships remain particularly vulnerable to the effects of rising food prices. Like much of the rest of the Global South, the food environment within South Africa is rapidly changing, with multinational food companies accounting for the majority of the market share^(9)^. Foods are increasingly being eaten away from home, with fast-food options increasing. The higher cost and limited availability of healthy foods make convenient healthy options unattainable for the majority of the population^(7,10,11)^. Additionally, the built environment in townships makes it difficult for low-income shoppers to select healthy foods, with less availability and poorer quality options available in low socioeconomic neighbourhoods^(12)^. As a result, cheap, energy-dense, ultra-processed and unhealthy food options are becoming the food of choice for many^(10)^.

Numerous studies associate the increased consumption of UPF with obesity and diet-related non-communicable diseases (NCD) like hypertension, diabetes, dyslipidaemia and certain cancers^(13–16)^. These diet-related NCD result in increased mortality levels, particularly in low- and middle-income countries, where the majority of these deaths occur^(17)^. Studies in South Africa have shown that foods are selected because they are cheap, filling and tasty, but not necessarily nutritious^(10,11)^. Consequently, NCD, such as diabetes and hypertension, are fast becoming the most burdensome diseases in the South African health system^(18,19)^. One in five women in South Africa is severely obese. Sixty-eight percent of women are overweight or obese, as are 31 % of men. Hypertension, overweight and obesity prevalence have been increasing since 1998^(20)^, and those living with obesity are more likely to suffer from disease multimorbidity^(21)^. On a macronutrient level, obese individuals may appear to be food secure, but on a micronutrient level, food and nutritional insecurities are prevalent^(22)^.

As UPF have been shown to be harmful to health, it is important to examine their intake when assessing dietary patterns and health of individuals and populations^(23)^. The Nova classification system, as a tool to identify UPF, has been used to assess dietary intake in a growing number of countries^(24–29)^. In 2015, the FAO included the Nova system in their guidelines on collecting food processing information from food surveys^(30)^, and a recent WHO report has used the Nova classification system to describe the increase in UPF intake in Latin America over the past decade^(31)^. Applying the Nova food classification system to food composition databases has been identified as a way to quantify the contribution of UPF to the food supply. This can assist in evaluating the quality of dietary intake in various population groups^(25)^. To our knowledge, only one other study has assessed dietary intake in relation to level of processing in South Africa^(32)^.

Therefore, the aims of this study are twofold. First, we seek to describe what share of the diet of low-income adults living in South Africa is comprised of UPF. Second, we seek to assess the adequacy of the diet of low-income adults in South Africa using WHO and World Cancer Research Fund dietary recommendations and evaluate potential associations with level of UPF consumption. Using international criteria to assess dietary intake creates the potential for comparison to other contexts, and analysing the intake of UPF in South Africa allows for better monitoring of the nutrition transition.

## Methods

Secondary analysis of dietary data from two purposively selected datasets collected in three low-income areas in South Africa (Langa and Khayelitsha in Cape Town and Mount Frere in the Eastern Cape) was undertaken. These two studies used different sampling strategies, but the same methodology and data collection instruments.

### Sampling procedures and data collection

Sampling procedures used in Khayelitsha and Mount Frere differed to those used in Langa, to meet their primary study objectives. The primary objective of the study conducted in Khayelitsha and Mount Frere was to assess the obesogenic food environment^(33)^, whilst the primary objective of the study conducted in Langa was to evaluate dietary changes as a result of the introduction of the health promotion levy (sugary beverage tax)^(34)^. Individuals were only included in the studies if they met the inclusion criteria, which included being between the ages of 18 and 50, residing in the study area, having knowledge about household food purchasing and consumption habits and providing informed consent to participate in the study.

In the study conducted in Khayelitsha and Mount Frere, 300 households were randomly sampled at each site in October and November 2017, with a 20 % oversampling margin to compensate for non-responders. A semi-purposive stratified sampling strategy was used to select research clusters. Four clusters per site were selected based on proximity to key features (transport hubs, supermarkets, main roads and living areas) as well as by housing type. Households in each quadrant were counted to determine an appropriate sampling interval for each cluster and a purposively selected starting point was chosen. In each household, one randomly selected individual was chosen as a respondent between the age of 18–50 years (respondents were stratified to ensure representation of gender). For the Langa sample, systematic door-to-door sampling was conducted in February and March 2018 throughout the entire area of Langa, with a target sample size of 2250 participants. One randomly selected consenting adult between the ages of 18–39 years old per household was included in the study.

All three study sites used the same questionnaires to collect dietary and general household information. The only exception was BMI derived from measured height and weight, which was only collected in Langa. The household socio-demographic questionnaire included previously validated socio-demographic questions, such as household characteristics, the household hunger scale^(35)^ and the lived poverty index^(36)^. A one-day standardised 24-h dietary recall was collected for each participant. Fieldworkers were extensively trained and fluent in languages spoken at the study sites. They used cellphones to digitally record socio-demographic data and paper-based questionnaires to complete the 24-h dietary recalls.

Participants from these primary studies were only included for secondary analysis if they had completed all the questionnaires and did not have any missing dietary intake data. In total, 2161 participants were included from Langa (85·3 % of respondents), 191 from Khayalitsha (61·2 % of respondents) and 169 from Mount Frere (51·2 % of respondents), for a total sample of 2521 adults.

### Data coding and analysis

All 24-h dietary recalls were coded by trained data capturers with a tertiary-level nutrition qualification. The South Africa Medical Research Council (SAMRC) food quantities^(37)^ and food composition tables^(38)^ (FCT) were used for coding. An extensive assumptions manual was developed to ensure assumptions were made in a standardised manner when necessary.

Demographic characteristics of study participants included age, sex, area of residence, household income and educational status. BMI was used to assess nutritional status. Dietary intake of study participants was assessed using intake reported in the 1-d 24-h dietary recall, and intake was examined by using mean, median and sd. Food and beverages were classified as UPF according to the Nova food classification system, a system that groups foods, for the purpose of public health policy, into four categories based on the purpose, nature and extent of industrial processing^(3,23)^. The four groups are (1) unprocessed or minimally processed foods, (2) processed culinary ingredients, (3) processed foods and (4) UPF^(3,39)^. As the share of energy intake coming from UPF was the outcome of interest, we classified products into two groups, UPF (Nova group 4) or not (Nova groups 1–3). Two registered dietitians independently applied the Nova classification to the foods and beverages consumed by study participants. Discrepancies between classifications were resolved by consulting with a third dietitian and reaching consensus. Quartiles of UPF consumption were created, based on the share of energy intake that UPF accounted for. Participants were considered to be low UPF consumers if they were within the lowest quartile of UPF consumption and high UPF consumers if they were in the highest quartile.

We used the Healthy Diet Indicator 2020 (HDI-2020)^(40)^ to assess components of diet quality. The HDI-2020 criteria is based on WHO^(41,42)^ and other international dietary recommendations. There are six components for which dietary intake should be restricted, and five components that should be encouraged, following the criteria shown in Table 1. More detail on the HDI-2020 can be read elsewhere^(40)^. When the data from the 24-h dietary recall alone were insufficient to assess whether or not the HDI-2020 criteria were met, the global diet quality questionnaire (DQ-Q) guidelines of the global diet quality score^(43)^ were used to identify products to include in the HDI-2020 criteria. Unfortunately, for nutrient-specific evaluations, missing values in the SAMRC FCT resulted in underreporting of intake for some nutrients in the analyses. This was particularly pronounced for total and added sugar. Please see the limitations section and Appendix A and B for more details. Additionally, there are no free sugar values in the food composition table, so added sugar was used as a proxy. The probability of meeting international dietary recommendations was compared between the lowest and highest quartiles of UPF consumers. For WHO guidelines that use the share of energy as the criteria, the contribution to share of total energy was calculated by quartile of UPF. For components where the guidelines were in grams, rather than share of total energy, the contribution per 1000 kJ was calculated.

**Table 1:** Global dietary recommendations assessed using the healthy diet indicator 2020 (HDI-2020)

		Dietary element Elements based on HDI-2020	Global recommendation	Reference source for guideline	Criteria for scoring (quantitative intake in one day)	Approach to coding and analysing data
**Dietary components to limit**	1	Total fat	<30 % total energy	World Health Organisation	<30 % total energy	Total fat identified and calculated from 24-h diet recall data
	2	Saturated fat	<10 % total energy	World Health Organisation	<10 % total energy	Saturated fat identified and calculated from 24-h diet recall data
	3	Salt (dietary sodium)	<5 g/d (<2000 mg sodium/d)	World Health Organisation	<2000 mg sodium	Dietary sodium identified and calculated from 24-h diet recall data
	4a	Free (added) sugars (24-h recall)	<10 % total energy	World Health Organisation	<10 % total energy	Added sugars identified and calculated from 24-h diet recall data
	4b	Free sugars (DQ-Q)	<10 % total energy	World Health Organisation	<10 % total energy	Free sugars identified using DQ-Q criteria Amount consumed calculated from 24-h diet recall data
	5	Processed meat	‘Consume very little, if any, processed meat’	World Cancer Research Fund	0 g	Processed meat identified using DQ-Q criteria Amount consumed calculated from 24-h diet recall data
	6	Unprocessed red meat	≤350–500 g/week	World Cancer Research Fund	≤71 g	Unprocessed red meat identified using DQ-Q criteria Amount consumed calculated from 24-h diet recall data
**Dietary components to encourage**	7	Fruits and vegetables	≥400 g/d	World Health Organisation	≥400 g	Fruits and vegetables identified using DQ-Q criteria Amount consumed calculated from 24-h diet recall data
	8	Beans and other legumes	‘A healthy diet contains…legumes’	World Health Organisation	>0 g	Beans and legumes identified using DQ-Q criteria Amount consumed calculated from 24-h diet recall data
	9	Nuts and seeds	‘A healthy diet contains… nuts’	World Health Organisation	>0 g	Nuts and seeds identified using DQ-Q criteria Amount consumed calculated from 24-h diet recall data
	10	Whole grains	‘A healthy diet contains…whole grains’	World Health Organisation	>0 g	Whole grains identified using DQ-Q criteria Amount consumed calculated from 24-h diet recall data
	11	Dietary fibre	>25 g/d	World Health Organisation	>25 g	Dietary fibre identified and calculated from 24-h diet recall data

DQ-Q, diet quality questionnaire.

We used STATA version 15 (StataCorp) to review, clean and analyse the data. Nutrient content was verified by identifying outliers, checking the original 24-h recalls and correcting the information when appropriate. Participants who consumed more than 20 000 kJ/d or < 400 kJ/d were excluded. Descriptive dietary intake statistics were performed and scores calculated for the household hunger scale^(35)^, lived poverty index^(36)^. The dietary diversity score was calculated from the dietary intake reported the 24-h recall and was assessed by evaluating the minimum dietary diversity for women^(44)^. The Mann–Whitney *U* test was used to compare differences in nutritional intake by gender. Due to the non-parametric nature of the data, quantile regression analysis was performed to assess differences in median nutrient intake by quartile of UPF consumption. Logistic regression analysis was performed to calculate the probability of low and high UPF consumers meeting WHO and other international dietary guidelines (using the HDI-2020 criteria). All models were adjusted for age, sex, household income and area of residence. For all analyses, a level of significance was assumed at *P* < 0·05.

## Results

### Demographics and dietary intake of low-income adults

Of the 2521 study participants, 68·1 % were female. Whilst 40·0 % of participants with anthropometric measurements (*n* 2024) had a normal weight, obesity prevalence levels were much higher in women, with 43·7 % (*n* 587) of women, and 7·9 % of men (*n* 54), living with obesity. The majority (86·2 %) of participants had not completed secondary education, and more than half of the participants had a monthly household income of R3000 (226 USD based on 2018 exchange rates) or less. Dietary diversity was only achieved for 24·3 % of female participants. Despite this, 86·4 % of participants reported little to no household hunger, and 60·5 % reported a low lived poverty index (see Table 2 Panel A).

**Table 2:** Share of total energy intake from ultra-processed foods (UPF) according to demographic characteristics^†^

		Panel A		Panel B		
		Distribution		Mean share of total energy intake from UPF		*P* value*
		*n*	%	%	se	
Sex	Male	804	31·89	38·97	0·85	0·062
	Female	1717	68·11	37·09	0·56	
Age	18–29	1453	57·64	40·24	0·62	<0·001*
	30–39	974	38·64	35·36	0·75	
	40–50	94	3·73	22·28	1·95	
Area	Khayelitsha	191	7·58	29·81	1·57	<0·001*
	Langa	2161	85·72	39·52	0·50	
	Mount Frere	169	6·70	23·13	1·68	
Household income (per month)	<R3001	1116	52·87	36·64	0·70	0·087
	R3001–R4000	312	14·78	36·72	1·31	
	R4001–R5000	211	10·00	38·22	1·74	
	R5001–R7500	207	9·81	38·86	1·58	
	R7501–R10000	137	6·49	39·87	2·11	
	>R10000	128	6·06	37·93	2·13	
Nutritional** status	Underweight	108	5·34	40·90	2·24	0·933
	Normal weight	809	39·97	39·85	0·83	
	Overweight	466	23·02	38·00	1·06	
	Obese	325	16·06	37·67	1·30	
	Severely obese	184	9·09	40·12	1·75	
	Morbidly obese	132	6·52	42·10	2·02	
Education level	No/min formal completed	123	4·90	30·17	2·13	0·002*
	Completed primary	2042	81·32	37·82	0·52	
	Completed secondary	346	13·78	39·42	1·33	
	Completed tertiary	0	0·00	–		
Lived poverty	Low (<0·51)	1491	60·51	37·89	0·62	0·023
	Low-med (0·51–1·0)	598	24·27	39·26	0·92	
	High-moderate (1·1–1·5)	214	8·69	38·08	1·67	
	High (>1·5)	161	6·53	30·75	1·73	
Household hunger	Little/no hunger	2169	86·41	37·83	0·51	0·469
	Moderate hunger	324	12·91	36·55	1·34	
	Severe hunger	17	0·68	39·05	6·24	
Minimum dietary diversity for women (MDD-W)‡	Achieved MDD-W	417	24·33	37·35	0·91	0·784
	Did not achieve MDD-W	1297	75·67	37·00	0·69	

* Regression analysis used to calculate *P* value. Level of significance assumed at *P* < 0.05.

† Missing values are due to anthropometry measurements only being taken in Langa (sample age 18–39 years; 2024 measurements taken).

‡ Minimum dietary diversity for women (MDD-W) includes only women, up to age of 49 years.

The mean energy intake was significantly higher amongst men than women (8551 kJ/d *v*. 7393 kJ/d; *P* < 0·001). This trend was also observed for most other nutrients. Men consumed significantly more total fat (59·7 g/d, *P* = 0·001) and saturated fat (16·8 g/d, *P* = 0·005) than women who consumed a mean 51·9 g/d and 14·9 g/d, respectively. The mean daily protein consumption was 10g higher in men than women (67·5 g *v*. 57·7 g, *P* < 0·001), whilst total sugar only differed by 2 g/d (64·3 g *v*. 62·5 g; *P* = 0·699). Interestingly, despite their energy intake being lower, women consumed significantly more added sugar than men (23·8 g/d *v*. 20·5 g/d; *P* < 0·001). They also consumed more whole grains and fruits and vegetables than men, although this was not statistically significant. The average fibre intake amongst participants was 17·4 g/d (see Table 3).

**Table 3:** Dietary intake of males and females aged 18–50 years in Langa, Khayelitsha and Mount Frere

	Female				Male				Total				*P* value
	Mean	s d	Median	IQR	Mean	sd	Median	IQR	Mean	sd	Median	IQR	
Energy (kJ)	7392·83	3123·21	6918·08	3894·77	8550·79	3549·63	8046·90	4332·76	7762·13	3308·87	7328·42	4074·97	<0·001**
Energy from UPF (kJ)	2873·91	2384·44	2415·70	2794·00	3464·03	2787·10	2984·65	3281·54	3062·11	2534·27	2595·88	3025·00	<0·001*
Protein (g)	57·65	29·69	52·43	35·52	67·47	34·05	62·12	40·67	60·78	31·48	55·27	36·35	<0·001*
Total fat (g)‡	51·91	34·53	45·12	41·54	59·71	42·85	49·76	45·19	54·40	37·55	46·30	43·18	0·001*
Saturated fat (g)	14·90	11·54	12·34	12·01	16·79	14·24	13·28	12·60	15·50	12·49	12·54	11·97	0·005*
MUFA (g)	17·35	13·10	14·28	14·29	19·97	17·12	15·24	15·66	18·18	14·55	14·60	14·63	0·003*
PUFA (g)	14·09	11·48	11·01	12·77	16·22	13·98	12·07	15·18	14·77	12·37	11·29	13·59	0·004*
Carbohydrate (g)	246·96	107·65	233·11	131·74	276·35	117·43	265·30	149·31	256·33	111·68	243·99	140·09	<0·001*
Total sugar (g)†	62·47	46·03	53·40	58·23	64·34	49·97	56·56	63·15	64·07	47·32	54·40	58·23	0·699
Added sugar (g)†	23·81	31·33	16·63	35·59	20·52	31·31	4·61	31·52	22·76	31·36	14·63	34·28	<0·001*
Dietary Na	1534·28	1301·44	1252·68	1215·13	1825·11	1515·98	1558·64	1565·59	1627·03	1379·88	1318·66	1336·48	<0·001*
Processed meat (g)	20·57	57·04	0·00	0·00	24·91	74·47	0·00	0·00	21·95	63·15	0·00	0·00	0·905
Unprocessed red meat (g)	22·31	68·54	0·00	0·00	35·53	100·73	0·00	0·00	26·53	80·44	0·00	0·00	0·008*
Dietary fibre (g)	16·82	10·48	14·79	11·27	18·62	11·35	16·66	12·74	17·40	10·80	15·30	11·78	<0·001*
Fruits and vegetables (g)	129·52	171·08	75·00	170·00	127·29	167·86	68·50	187·00	128·81	170·03	75·00	175·00	0·186
Beans and other legumes (g)	4·09	23·92	0·00	0·00	5·43	30·23	0·00	0·00	4·52	26·10	0·00	0·00	0·447
Nuts and seeds (g)	1·71	7·94	0·00	0·00	2·79	11·36	0·00	0·00	2·06	9·18	0·00	0·00	0·080
Whole grains (g)	29·47	99·37	0·00	0·00	23·67	88·09	0·00	0·00	27·62	95·94	0·00	0·00	0·133

* Level of significance assumed at *P* < 0.05. Mann–Whitney *U* test used to analyse level of significant difference between males and females.

† For nutrient-specific evaluations, missing values in the South African food composition table resulted in an underestimate of values, which was particularly pronounced for total and added sugar (see Appendix Tables A and B for details).

‡ Trans-fats excluded from all analysis due to insufficient data in the South African food composition table.

### Ultra-processed product intake

The percentage of total energy intake from UPF was similar amongst men and women (39·0 % and 37·1 %, respectively, *P* = 0·062). UPF intake accounted for a significantly larger share of dietary intake amongst younger consumers, contributing 40·2 % of daily energy intake amongst 18–29-year-olds, and 22·3 % of intake amongst 40–50-year-olds (*P* < 0·001). Household income was not associated with the proportion of UPF consumed (*P* = 0·087), as those with the lowest household income (<R3001/d) consumed a similar proportion of UPF to the highest income households (>R10 000/d), at 36·7 % and 37·9 % of total daily intake, respectively. Those without any formal education consumed significantly lower amounts of UPF (30·2 %) than those who had completed primary (37·8 %) and secondary (39·4 %) education (*P* = 0·002) (see Table 2 Panel B).

**Fig. 1:**
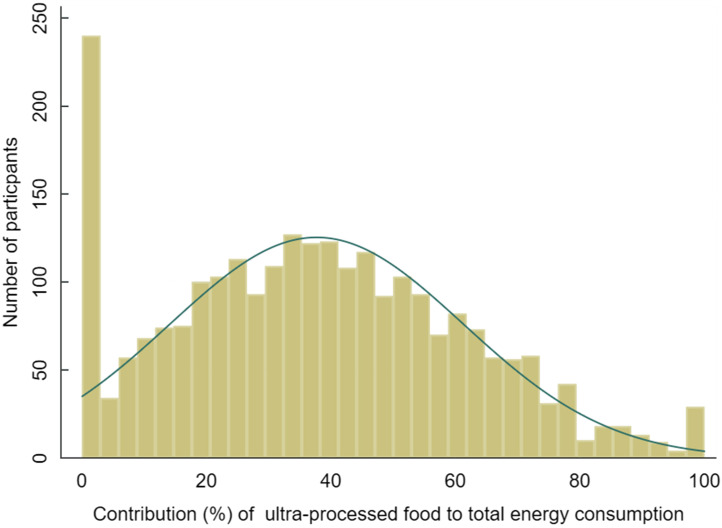
Distribution of the share of UPF to total energy intake. UPF, ultra-processed foods.

Figure 1 shows the distribution of the share of UPF to total energy intake amongst study participants. Very few participants (7·6 %, *n* 192) reported that they did not consume any UPF in the previous day. There were clear gradients with respect to nutrient intake, when analysed by quartile of share of energy from UPF. The highest quartile of UPF consumers consumed a median 10264 kJ/d of total energy (60·3 % of which was accounted for by UPF intake), whilst the lowest quartile consumed a median 5605 kJ/d (of which 7·8 % was attributed to UPF). The same significant trend by quartile for median intake was observed for total fat, saturated fat, total sugar and Na, with the highest UPF consumers consuming the largest quantities of these nutrients of concern linked with NCD and obesity. Added sugar intake also increased by UPF quartile, but the difference between quartiles was NS. Interestingly, median total fibre and fruit and vegetable intake also increased by quartile of UPF consumers, although the increase was NS for fruits and vegetables. In both of these groups, despite the increase in absolute terms, the opposite trend, which was significant, was observed for g/1000kJ, with the contribution decreasing with each ascending quartile (whilst Na had the opposite trend) (Table 4).

**Table 4:** Dietary intake by quartile of share of energy from ultra-processed foods (UPF) for adults aged 18–50 years in Langa, Khayalitsha and Mount Frere

				UPF							*P* value	Share of total energy								*P* value
		*N* †	Q1		Q2		Q3		Q4			Q1 UPF		Q2 UPF		Q3 UPF		Q4 UPF		
			Median	se	Median	se	Median	se	Median	se		%	se	%	se	%	se	%	se	
Dietary components to limit	Total energy (kJ/d)	2111	5605·07	144·28	6485·22	127·44	7604·26	137·92	10264·25	155·25	<0·001*	–		–		–		–		–
	Energy from UPF (kJ/d)	2111	529·87	40·53	1931·93	26·70	3281·72	38·36	5803·44	105·89	<0·001*	7·75	0·67	30·58	0·69	44·47	0·77	60·29	0·88	<0·001*
	Total fat (g/d)	2111	28·24	1·06	38·06	0·98	50·23	0·96	79·64	2·09	<0·001*	20·44	0·46	23·07	0·56	25·53	0·39	29·44	0·58	<0·001*
	Saturated fat (g/d)	2111	7·57	0·35	10·59	0·31	13·67	0·37	21·74	0·65	<0·001*	5·27	0·15	6·03	0·14	6·84	0·16	8·28	0·22	<0·001*
	Total sugar‡ (g/d)	2111	31·24	1·57	48·16	1·78	63·63	1·88	85·94	2·73	<0·001*	9·15	0·42	13·18	0·50	14·66	0·40	14·53	0·42	<0·001*
	Added sugar‡ (g/d)	2111	6·48	0·95	12·82	1·62	12·88	1·71	21·34	1·80	0·331	1·93	0·28	3·23	0·39	2·90	0·39	3·36	0·35	0·016*
												Contribution (mg or g) per 1000kJ energy								
	Dietary Na (mg/d)	2111	466·87	28·61	1073·50	21·27	1584·16	34·82	2624·83	57·66	<0·001*	83·38	5·71	172·32	3·27	215·07	4·69	262·35	5·89	<0·001*
Dietary components to encourage	Dietary Fibre (g/d)	2111	12·00	0·43	14·45	0·46	15·74	0·30	19·50	0·42	<0·001*	2·26	0·06	2·20	0·05	2·11	0·04	1·89	0·04	<0·001*
	Fruits and Vegetables (g/d)	2111	70·85	5·77	75·85	6·73	72·85	5·96	70·85	5·20	0·098	13·47	1·21	12·29	1·22	9·74	0·76	7·13	0·51	0·004*

* Adjusted for age, sex, household income and area of residence. Level of significance assumed at *P* < 0.05.

† 410 participants excluded from all analysis due to missing data on household income.

‡ For nutrient-specific evaluations, missing values in the South African food composition table resulted in an underestimate of values, which was particularly pronounced for total and added sugar (see Appendix Tables A and B for details).

Quantile regression analysis performed due to non-parametric data to assess differences in intake by quartile of UPF consumption. Quartiles of UPF consumption were created based on the share of absolute energy intake that UPF accounted for. Participants were considered to be low UPF consumers if they were within the lowest quartile of UPF consumption, and high UPF consumers if they were in the highest quartile of UPF consumption.

Analysis of processed meats, unprocessed red meat, beans and other legumes, nuts and seeds and whole grains excluded from analysis due to low number of participants consuming these dietary components and small cell counts not permitted for quantile regression.

### Adequacy of the diet based on international recommendations

In Table 5, Panel A shows that overall, very few participants met international recommendations for dietary components that are beneficial to health. Only 7·0 % of participants met the WHO recommended intake of 400 g of fruit and vegetables per day in the previous day. The mean intake amongst the 1963 participants who did not meet the guideline was 93·0 g/d. Similarly, low numbers of participants met the protective recommendations for frequent consumption of beans and other legumes, nuts and seeds and whole grains (with 4·6 %, 7·3 % and 15·6 % meeting each respective guideline). Slightly more (18·8 %) participants met the recommended intake of 25 g or more of fibre per day, although the mean intake amongst those who did not meet it remained low, at 13·9 g/d

**Table 5:** Using the healthy diet indicator 2020 to assess the probability of low and high ultra-processed foods (UPF) consumers meeting WHO and other international dietary guidelines

					Panel A								Panel B				
		Dietary element	Criteria for scoring (quantitative intake in one day)	*n*†	Meets guideline				Does not meet guideline								*P*-value*
					*n*	%	Mean	s e	*n*	%	Mean	se	Predicted probability of meeting guideline if low UPF consumer‡		Predicted probability of meeting guideline if high UPF consumer‡		
													%	se	%	se	
Dietary components to limit	1	Total fat	<30 % total energy	2111	1437	68·07	39·44	0·59	674	31·93	85·49	1·63	78·59	1·80	52·40	2·22	<0·001*
	2	Saturated fat	<10 % total energy	2111	1698	80·44	11·63	0·17	413	19·56	31·06	0·82	88·89	1·38	66·34	2·11	<0·001*
	3	Dietary Na	<2000 mg sodium	2111	1535	72·71	1001·80	14·24	576	27·29	3290·02	66·29	97·88	0·61	25·00	1·94	<0·001*
	4a	Free (added) sugars (24-h recall)	<10 % total energy	2111	1732	82·05	12·86	0·40	379	17·95	67·00	2·05	79·77	1·73	83·71	1·67	0·109
	4b	Free sugars (DQ-Q)	<10 % total energy	2111	1209	57·27	22·09	0·83	902	42·73	23·24	1·13	79·76	1·80	40·35	2·13	<0·001*
	5	Processed meat	0 g	2111	1680	79·58	0·00	0·00	431	20·42	103·63	5·01	94·21	0·98	60·86	2·19	<0·001*
	6	Unprocessed red meat	≤71 g	2111	1834	86·88	1·96	0·26	277	13·12	189·25	8·09	87·72	1·46	84·66	1·56	0·156
Dietary components to encourage	7	Fruits and vegetables	≥400 g	2111	148	7·01	589·65	18·84	1963	92·99	93·86	2·26	7·49	1·19	6·82	1·07	0·678
	8	Beans and other legumes	>0 g	2111	98	4·64	99·62	7·96	2013	95·36	0·00	0·00	7·34	1·16	2·95	0·73	0·002*
	9	Nuts and seeds	>0 g	2111	153	7·25	29·27	1·65	1958	92·75	0·00	0·00	1·46	0·52	12·26	1·48	<0·001*
	10	Whole grains	>0 g	2111	329	15·59	167·25	9·58	1782	84·41	0·00	0·00	10·15	1·31	17·32	1·70	0·001*
	11	Dietary fibre	>25 g	2111	396	18·76	34·72	0·54	1715	81·24	13·91	0·14	15·06	1·56	26·56	1·96	<0·001*

UPF,ultra-processed foods.

* Logistic regression analysis performed to calculate the probability of meeting dietary guidelines by quartile of UPF intake. Adjusted for age, sex, household income and area of residence. Level of significance assumed at *P* < 0.05.

† 410 participants excluded from all analysis due to missing data on household income. For nutrient-specific evaluations, missing values in the South African food composition table resulted in an underestimate of compliance with guidelines. This was particularly pronounced for total and added sugar (see Appendix A and B for more details).

‡ Low UPF consumers are those with the lowest quartile of UPF consumption, and high UPF consumers are those with the highest quartile of UPF consumption.

At least 50 % of participants met the recommendations for all nutrients to limit. Sixty-eight percent of participants met the recommendation of consuming < 30 % of their total energy intake from fat, 80·4 % consumed < 10 % of their total energy intake from saturated fat per day and 72·7 % consumed < 2000 mg Na per day. No processed meat was consumed by 79·6 % of participants on the previous day, and 86·9 % of participants did not consume excessive amounts of red meat. Although free and added sugar intakes were difficult to assess (see the methods and limitation section for more details), depending on whether intake was assessed using the free sugar criteria from the DQ-Q or the 24-h recall data for added sugar, between 57·3 % and 82·1 % met the recommendation of < 10 % of total energy, respectively (Table 5 Panel A).

### Alignment of international dietary recommendations and the Nova system in assessing dietary inadequacy

In Table 5, Panel B shows the predicted probabilities of meeting international guidelines by level of UPF consumption (high *v*. low). For dietary recommendations that consider the share of total energy (total fat, saturated fat and free sugar), high UPF consumers were significantly less likely to meet the recommendation than low UPF consumers. The only exception was free sugar intake when using added sugar from the 24-h recall, which did not have a significant difference. High UPF consumers were also significantly more likely to have excessive Na and processed meat intake and insufficient bean and legume consumption. However, high UPF consumers were significantly more likely to meet the recommendations for nuts and seeds, wholegrain and fibre intake. No significant differences were observed between high and low UPF consumers for fruit and vegetable intake or unprocessed red meat consumption.

## Discussion

Despite the participants being low-income adults living in South Africa, most participants reported either low or low-medium levels of lived poverty, and only 13·6 % reported moderate to severe hunger. These findings are somewhat aligned with findings from studies conducted amongst low-income South Africans^(45,46)^, although since undertaking this study (data collected in 2017 and 2018), levels of lived poverty and food insecurity have worsened in South Africa^(45,46)^. Only 24·3 % of women met the criteria for minimum dietary diversity, indicating that although they might not report high levels of hunger, the diet is not nutritionally diverse, and is lacking in micronutrients. This is supported in that, for all components identified in the HDI-2020 to be protective for health, less than 20 % of participants consumed adequate amounts. The number of participants who met the fibre recommendation (18·8 %) was similar to the number of participants who met the wholegrain recommendation (15·6 %), which also contributes towards fibre intake. These protective foods are often costly, resulting in cheaper, more filling and unhealthier alternatives being selected instead^(10)^.

While most participants met the recommendations for total fat, saturated fat and Na intake, examining nutrient intake based on energy consumed from UPF reveals that disparities exist in the healthfulness of participants diets. Those who consumed the most UPF also consumed the most energy and dietary components that are recommended to be restricted, except for red meat where no significant difference was observed in the two groups. Numerous studies have linked high UPF consumption to poor health outcomes^(47)^. While we did not look at specific health outcomes, we found a clear positive gradient of association between share of energy from UPF and nutrients of concern and an inverse association between share of energy from UPF and dietary components to encourage. Consequently, the probability of meeting international dietary guidance is higher among those who are in the lowest UPF consumption quantile compared with those who are on the highest quantile of UPF consumption. Given the findings of this study, and others around the world that have found high and increasing intake of UPF^(15)^, the WHO should consider introducing a recommendation regarding the maximum recommended share that UPF should contribute to total energy (similar to the guidelines it has developed for saturated fat or free sugar).

The high level of UPF consumption reported amongst low-income South Africans in our study supports literature that indicates that the nutrition transition is advanced in South Africa^(48)^, which typically goes hand in hand with the proliferation of UPF, and increasing levels of obesity and nutrition-related NCD^(15)^. This study highlights the need for the South African Government to implement better strategies to protect South Africans against the proliferation of UPF, and more importantly to protect low-income South Africans who are most vulnerable to the economic shocks of poor health outcomes from undue influence towards UPF consumption. Recent studies in South Africa have found that 76 % of packaged foods sold in South African supermarkets are UPF^(49)^, and that more shelf space in stores is allocated to unhealthy products than healthy products^(50)^ leaving little room for consumers to make healthy food choices. There is a need to ensure that healthy and nutritious foods are readily available, affordable and desirable to consumers, including low-income people, and that unhealthy UPF are less predominantly the food of choice.

A policy that the South African National Department of Health is currently considering and could contribute to an improved food environment is mandatory front-of-package warning labels^(51)^. These labels inform consumers about products containing excessive amounts of nutrients of concern and can in turn be used to inform further regulations, such as marketing restrictions (e.g. barring two-for-the-price-of-one specials, promotions to win prizes, advertisements to children, etc.), restricting these products in schools or at point-of-sale in supermarkets where consumers are more likely to make rash decisions. Such policies have already been or are soon to be implemented in Chile, Mexico, Peru, Israel, Singapore and the United Kingdom. Additionally, measures similar to the Health Promotion Levy (a tax on sugary beverages which has been found to be effective in South Africa)^(34,52)^ could be considered for products that carry a front-of-package warning label. Revenue raised could be used to subsidise the price of healthier food choices. In the same way that unhealthy UPF should be restricted, the consumption of healthy fresh foods should be encouraged.

### Limitations and assumptions

This study has a number of limitations that need to be kept in mind when interpreting the data. First, only two of the nine provinces of South Africa were included, which limits the generalisability of findings. Data from two different studies were included. Although similar methodologies, standardised training and the same questionnaires were used for both studies, sampling strategies differed between the studies, some of the fieldworkers differed and study participants were not exactly the same. The sample size also differed significantly by area. To try and account for this, regression analysis results were adjusted for area of residence, age, sex and household income.

Second, seasonality has been found to influence dietary intake^(53)^. Although three seasons were included during data collection (summer and autumn in Langa and spring in Khayalitsha and Mount Frere), it is possible that results may have differed had all four seasons been included.

Third, when collecting dietary data, one needs to take self-reported bias as well as social desirability bias into account. Dietary assessment was based on a single-day 24-h recall due to the available secondary data and did not capture intra-person day-to-day variation in intake. The distribution of intake would have been better accounted for with two or more 24-h recalls per participant or the inclusion of a quantified FFQ for a subset of the sample; however, the large sample size of this study allows for sufficiently accurate means with a single-day recall^(53)^. Despite the one day of recall data likely resulting in a wider distribution of intake due to more measurement error, the rank ordering of quartiles is still appropriate, assuming measurement error is random. The observations in the top 25th percentile would very likely be the same, even with multiple days of recall.

Fourth, there were missing values in the SAMRC FCT, particularly for total and added sugar. Thus when the SAMRC FCT was applied to the intake data, we found that 19·4 % and 30·6 % of food items reported consumed were missing total and added sugar values, respectively. More than 50 % of these missing values were UPF products, and missingness was greater among UPF products than among all products. There were no missing values for energy, and five or less percent of missing values for total fat, saturated fat, Na and fibre, and thus the degree of underestimation of intake is higher for total and added sugar. Food groups where more than 40 % of consumed products were UPF included soups, sauces and seasonings; beverages; sugars, syrups and sweets and other products. These food groups tended to have higher numbers of missing values originating from UPF products than products that were not UPF (although this varied by nutrient and food group). As such, the findings presented in this paper regarding the share of nutrients of concern to discourage are likely conservative in terms of the association between the percentage of UPF consumed and nutrient outcomes (see Appendix A and B for more details). Additionally, the SAMRC FCT does not have brand level nutritional information (nor was this captured in the 24-h recalls). The nutritional composition of packaged UPF can differ significantly from one brand to another. However, the SAMRC FCT is the only South African specific FCT available, and thus remains the most appropriate FCT to use currently.

Fifth, assumptions needed to be made when classifying products according to the Nova food classification categories. As the secondary dietary data used for this study were not detailed, certain assumptions such as whether products were home-made or shop bought needed to be made. Although steps were put in place to limit classification errors, it is possible that some products were incorrectly classified. Little to no analysis of UPF using 24-h recall data has been conducted in South Africa previously, so despite the limitations with the dietary data, this study provides a baseline assessment of UPF consumption amongst low-income people living in South Africa.

### Conclusion

The nutrition transition is advanced, and UPF consumption is prevalent amongst low-income consumers in South Africa. UPF contribute disproportionately to energy intake, especially amongst those with the highest UPF consumption, and these high UPF consumers consume larger amounts of nutrients associated with increased NCD risk. Compared with low UPF consumers, high UPF consumers have higher overall energy consumption, higher Na, sugar and fat intake and are less likely to meet WHO recommendations for nutrients to limit. Most low-income adults living in South African assessed in this study consumed insufficient protective dietary components such as fibre, legumes, fruits and vegetables and had insufficient dietary diversity. Policy measures are urgently needed in South Africa to protect against the proliferation of harmful UPF and to promote and enable the consumption of whole and less processed foods. These measures could include a mandatory front-of-package warning label, marketing restrictions, restrictions on the types of foods available in schools. Additionally, a tax on unhealthy products could be used to promote the consumption of healthier food options by using revenue raised to subsidise the cost of healthy food options. There is an urgent need to realign the food system in South Africa and make healthy options achievable for all.

## Supporting information

Frank et al. supplementary material Frank et al. supplementary material

## Supplementary material

For supplementary material accompanying this paper visit https://doi.org/10.1017/S1368980023002811

## References

[1] Imamura F , Micha R , Khatibzadeh S et al. (2015) Dietary quality among men and women in 187 countries in 1990 and 2010: a systematic assessment. Lancet Glob Heal 3, e132–e142. 10.1016/S2214-109X(14)70381-X PMC4342410 25701991

[2] Moodie R , Stuckler D , Monteiro C et al. (2013) Profits and pandemics: prevention of harmful effects of tobacco, alcohol, and ultra-processed food and drink industries. Lancet 381, 670–679. 23410611 10.1016/S0140-6736(12)62089-3

[3] Monteiro CA , Cannon G , Moubarac JC et al. (2017) The UN decade of nutrition, the NOVA food classification and the trouble with ultra-processing. Public Health Nutr 21, 5–17. 28322183 10.1017/S1368980017000234 PMC10261019

[4] Poti JM , Braga B & Qin B (2017) Ultra-processed food intake and obesity: what really matters for health—processing or nutrient content? Curr Obes Rep 6, 420–431. 29071481 10.1007/s13679-017-0285-4 PMC5787353

[5] Monteiro CA , Moubarac JC , Cannon G et al. (2013) Ultra-processed products are becoming dominant in the global food system. Obes Rev 14, 21–28. 24102801 10.1111/obr.12107

[6] Stuckler D , McKee M , Ebrahim S et al. (2012) Manufacturing epidemics: the role of global producers in increased consumption of unhealthy commodities including processed foods, alcohol, and tobacco. PLoS Med 9, e1001235. 22745605 10.1371/journal.pmed.1001235 PMC3383750

[7] World Bank (2023) Poverty and Equity Brief. Africa Eastern & Southen. South Africa. https://www.worldbank.org/en/topic/poverty/publication/poverty-and-equity-briefs (accessed October 2023).

[8] Statistics South Africa (2022) Quarterly labour force survey. Quarter 4. Pretoria: Statistics South Africa. https://www.statssa.gov.za/publications/P0211/P02114thQuarter2022.pdf (accessed October 2023).

[9] Igumbor EU , Sanders D , Puoane TR et al. (2012) “Big food,” the consumer food environment, health, and the policy response in South Africa. PLoS Med 9, e1001253. 22802733 10.1371/journal.pmed.1001253 PMC3389030

[10] Temple NJ & Steyn NP (2011) The cost of a healthy diet: a South African perspective. Nutr 27, 505–508. 10.1016/j.nut.2010.09.005 21074973

[11] Statistics South Africa (2015) Technical Report: Methodological report on rebasing of national poverty lines and development on pilot provincial poverty lines. Pretoria: Statistics South Africa. http://beta2.statssa.gov.za/publications/Report-03-10-11/Report-03-10-11.pdf (accessed October 2023).

[12] Odunitan-Wayas F , Okop K , Dover R et al. (2018) Food purchasing characteristics and perceptions of neighborhood food environment of South Africans living in low-, middle- and high-socioeconomic neighborhoods. Sustainability 10, 4801.

[13] Hall KD , Ayuketah A , Brychta R et al. (2019) Ultra-processed diets cause excess calorie intake and weight gain: an inpatient randomized controlled trial of ad libitum food intake. Cell Metab 30, 67–77.e3. 31105044 10.1016/j.cmet.2019.05.008 PMC7946062

[14] Monteiro CA , Moubarac JC , Levy RB et al. (2018) Household availability of ultra-processed foods and obesity in nineteen European countries. Public Health Nutr 21, 18–26. 28714422 10.1017/S1368980017001379 PMC10260838

[15] Baker P , Machado P , Santos T et al. (2020) Ultra-processed foods and the nutrition transition: global, regional and national trends, food systems transformations and political economy drivers. Obes Rev 21, 1–22. 10.1111/obr.13126 32761763

[16] Chen X , Zhang Z , Yang H et al. (2020) Consumption of ultra-processed foods and health outcomes: a systematic review of epidemiological studies. Nutr J 19, 86. 32819372 10.1186/s12937-020-00604-1 PMC7441617

[17] World Health Organisation (2010) Global status report on noncommunicable diseases. Geneva: WHO. https://digitallibrary.un.org/record/706319?ln=en (accessed October 2023).

[18] Wicks M , Wright H & Wentzel-Viljoen E (2017) Restricting the marketing of foods and non-alcoholic beverages to children in South Africa: are all nutrient profiling models the same? Br J Nutr 116, 2150–2159. 10.1017/S0007114516004244 28088922

[19] Roomaney RA , van Wyk B , Turawa EB et al. (2021) Multimorbidity in South Africa: a systematic review of prevalence studies. BMJ Open 11, e048676. 10.1136/bmjopen-2021-048676 PMC8496399 34615675

[20] Statistics South Africa (2017) Key Indicator Report: South African Demographic and Health Survey (SADHS). Pretoria: Statistics South Africa. https://www.statssa.gov.za/publications/Report%2003-00-09/Report%2003-00-092016.pdf (accessed October 2023).

[21] Roomaney RA , van Wyk B , Cois A et al. (2022) One in five South Africans are multimorbid: an analysis of the 2016 demographic and health survey. PLoS One 17, e0269081. 35617298 10.1371/journal.pone.0269081 PMC9135225

[22] Mchiza Z , Steyn N , Hill J et al. (2015) A review of dietary surveys in the adult South African population from 2000 to 2015. Nutrients 7, 8227–8250. 26404371 10.3390/nu7095389 PMC4586583

[23] Moubarac JC , Parra DC , Cannon G et al. (2014) Food classification systems based on food processing: significance and implications for policies and actions: a systematic literature review and assessment. Curr Obes Rep. 3, 256–272. 26626606 10.1007/s13679-014-0092-0

[24] Monteiro CA , da Costa Louzada ML , Steele E et al. (2018) Ultra-processed food consumption and chronic non-communicable diseases-related dietary nutrient profile in the UK (2008–2014). Nutrients 10, 587. 29747447 10.3390/nu10050587 PMC5986467

[25] O’Halloran SA , Lacy KE , Grimes CA et al. (2017) A novel processed food classification system applied to Australian food composition databases. J Hum Nutr Diet 30, 534–541. 28124481 10.1111/jhn.12445

[26] Juul F & Hemmingsson E (2015) Trends in consumption of ultra-processed foods and obesity in Sweden between 1960 and 2010. Public Health Nutr 18, 3096–3107. 25804833 10.1017/S1368980015000506 PMC10277202

[27] Solberg SL , Terragni L & Granheim SI (2016) Ultra-processed food purchases in Norway: a quantitative study on a representative sample of food retailers. Public Health Nutr 19, 1990–2001. 26695872 10.1017/S1368980015003523 PMC10271152

[28] Moubarac JC , Martins APB , Claro RM et al. (2013) Consumption of ultra-processed foods and likely impact on human health. Evidence from Canada. Public Health Nutr 16, 2240–2248. 23171687 10.1017/S1368980012005009 PMC10271334

[29] da Costa Louzada ML , Baraldi LG , Steele EM et al. (2015) Consumption of ultra-processed foods and obesity in Brazilian adolescents and adults. Prev. Med. 81, 9–15. 26231112 10.1016/j.ypmed.2015.07.018

[30] Food and Agriculture Organization (2015) Guidelines on the collection of information on food processing through food consumption surveys. Rome: FAO. https://www.fao.org/documents/card/es/c/a7e19774-1170-4891-b4ae-b7477514ab4e/ (accessed October 2023).

[31] Pan American Health Organization (2015) Ultra-processed food and drink products in Latin America: Trends, impact on obesity, policy implications. Washington, DC: PAHO. https://iris.paho.org/bitstream/handle/10665.2/7699/9789275118641_eng.pdf (accessed Oct 2023).

[32] Jacobs I , Taljaard-Krugell C , Wicks M et al. (2022) Degree of food processing and breast cancer risk in black urban women from Soweto, South African: the SABC study. Br J Nutr 128, 2278–2289. 35109954 10.1017/S0007114522000423 PMC9346100

[33] Kroll F , Swart EC , Annan RA et al. (2019) Mapping obesogenic food environments in South Africa and Ghana: correlations and contradictions. Sustainability 11, 3924.

[34] Essman M , Taillie LS , Frank T et al. (2021) Taxed and untaxed beverage intake by South African young adults after a national sugar-sweetened beverage tax: a before-and-after study. PLOS Med 18, e1003574. 34032809 10.1371/journal.pmed.1003574 PMC8148332

[35] Ballard T , Coates J , Swindale A et al. (2011) Household Hunger Scale : Indicator Definition, Measurement Guide. Washington, DC: FANTA, FHI 360. https://www.fantaproject.org/sites/default/files/resources/HHS-Indicator-Guide-Aug2011.pdf (accessed October 2023).

[36] Mattes R , Dulani B & Gyimah-Boadi E (2016) Africa’s growth dividend? Lived poverty drops across much of the continent. Policy Paper no. 29. Cape Town: Afrobarometer. https://afrobarometer.org/sites/default/files/publications/Policy%20papers/ab_r6_policypaperno29_lived_poverty_declines_in_africa_eng.pdf (accessed October 2023).

[37] Langenhoven M , Conradie P , Wolmarans P et al. (1991) Medical Research Council Food Quantities Manual, 2nd ed. Cape Town: South African Medical Research Council.

[38] Medical Research Council (2017) Food Composition Tables for South Africa, 5th ed. Cape Town: Medical Research Council.

[39] Monteiro CA , Cannon G , Levy R et al. (2016) NOVA. The star shines bright. World Nutr 7, 28–38.

[40] Herforth AW , Wiesmann D , Martínez-Steele E et al. (2020) Introducing a suite of low-burden diet quality indicators that reflect healthy diet patterns at population level. Curr Dev Nutr 4, 1–14. 33344879 10.1093/cdn/nzaa168 PMC7723758

[41] World Health Organization (2012) Guideline: Sodium intake for adults and children. Geneva: WHO. https://www.who.int/publications/i/item/9789241504836 (accessed October 2023). 23658998

[42] World Health Organization (2015) Guideline: Sugars intake for adults and children. Geneva: WHO. https://www.who.int/publications/i/item/9789241549028 (accessed October 2023). 25905159

[43] Bromage S , Batis C , Bhupathiraju SN et al. (2021) Development and validation of a novel food-based global diet quality score (GDQS). J Nutr 151, 75S–92S. 34689200 10.1093/jn/nxab244 PMC8542096

[44] Food and Agriculture Organization (2021) Minimum dietary diversity for women. Rome: FAO. https://www.fao.org/nutrition/assessment/tools/minimum-dietary-diversity-women/en/ (accessed October 2023).

[45] Mattes R (2020) *Lived poverty on the rise: Decade of living-standard gains ends in Africa*. Cape Town: Afrobarometer. https://www.afrobarometer.org/sites/default/files/publications/Policy%20papers/ab_r7_pap13_lived_poverty_on_the_rise_in_africa_1.pdf (accessed October 2023).

[46] Statistics South Africa (2019) *Measuring food security in South Africa: Applying the food insecurity experience scale*. Report 03-00–19. Pretoria: Statistics South Africa. http://www.statssa.gov.za/publications/Report-03-00-19/Report-03-00-192020.pdf (accessed October 2023).

[47] Popkin BM , Barquera S , Corvalan C et al. (2021) Towards unified and impactful policies to reduce ultra-processed food consumption and promote healthier eating. Lancet Diabetes Endocrinol. 9, 462–470. 33865500 10.1016/S2213-8587(21)00078-4 PMC8217149

[48] Haggblade S , Duodu KG , Kabasa JD et al. (2016) Emerging early actions to bend the curve in Sub-Saharan Africa’s nutrition transition. Food Nutr Bull 37, 219–241. 27036627 10.1177/0379572116637723

[49] Frank T , Thow AM , Ng SW et al. (2021) A fit-for-purpose nutrient profiling model to underpin food and nutrition policies in South Africa. Nutrients 13, 1–22. 10.3390/nu13082584 PMC8401225 34444744

[50] Odunitan-Wayas FA , Okop KJ , Dover RVH et al. (2021) Food purchasing behaviour of shoppers from different South African socio-economic communities: results from grocery receipts, intercept surveys and in-supermarkets audits. Public Health Nutr. 24, 665–676. 10.1017/S1368980020001275 PMC11574871 32611454

[51] Bopape M , Taillie LS , Frank T et al. (2021) South African consumers’ perceptions of front-of-package warning labels on unhealthy foods and drinks. PLoS One 16, e0257626. 34570825 10.1371/journal.pone.0257626 PMC8475997

[52] Hofman KJ , Stacey N , Swart EC et al. (2021) South Africa’s health promotion levy: excise tax findings and equity potential. Obes Rev 22, 1–7. 10.1111/obr.13301 PMC8349837 34060197

[53] Willett W (2012) Nutritional Epidemiology, 3rd ed. Oxford: University Press.

