# Supplementary material for: Dietary intake of low-income adults in South Africa: ultra-processed food consumption a cause for concern
Source: Public Health Nutr. 2024 Jan 11;27(1):e41. doi: 10.1017/S1368980023002811 (PMC10882538; doi:10.1017/S1368980023002811)
Supplement: Frank et al. supplementary material [file S1368980023002811sup001.docx]

**Appendix A.** Total, and ultra-processed foods (UPF) products consumed by participants with missing nutrient values in the food composition table, by food group

|  | Number of unique food codes in dataset | | Number of products consumed by participants | | Number of products with missing total fat values | | Number of products with missing saturated fat values | | Number of products with missing total sugar values | | Number of products with missing added sugar values | | Number of products with missing sodium values | | Number of products with missing fibre values | |
| --- | --- | --- | --- | --- | --- | --- | --- | --- | --- | --- | --- | --- | --- | --- | --- | --- |
| **Food group** | **UPF**  **n (%)** | **Total**  **n (%)** | **UPF**  **n (%)** | **Total**  **n (%)** | **UPF**  **n (%)** | **Total**  **n (%)** | **UPF**  **n (%)** | **Total**  **n (%)** | **UPF**  **n (%)** | **Total**  **n (%)** | **UPF**  **n (%)** | **Total**  **n (%)** | **UPF**  **n (%)** | **Total**  **n (%)** | **UPF**  **n (%)** | **Total**  **n (%)** |
| **Cereal and cereal products** | 58 (61.05) | 95 (100.00) | 2759 (42.77) | 6451 (100.00) | 0  (0.00) | 0  (0.00) | 41  (97.62) | 42  (0.65) | 1993 (54.87) | 3632 (56.30) | 2069 (53.35) | 3878 (60.11) | 1  (4.76) | 21  (0.33) | 0  (0.00) | 1  (0.02) |
| **Vegetables** | 4  (3.54) | 113 (100.00) | 44  (1.03) | 4266 (100.00) | 0  (0.00) | 233 (5.46) | 0  (0.00) | 86  (2.02) | 0  (0.00) | 11  (0.26) | 13  (3.78) | 344 (8.06) | 0  (0.00) | 1  (0.02) | 0  (0.00) | 0  (0.00) |
| **Fruits** | 0  (0.00) | 32 (100.00) | 0  (0.00) | 612 (100.00) | 0  (0.00) | 18  (2.94) | 0  (0.00) | 91 (14.87) | 0  (0.00) | 0  (0.00) | 0  (0.00) | 10  (1.63) | 0  (0.00) | 0  (0.00) | 0  (0.00) | 0  (0.00) |
| **Legumes and legume products** | 2  (18.18) | 11 (100.00) | 5  (4.03) | 124 (100.00) | 0  (0.00) | 0  (0.00) | 5  (62.50) | 8  (6.45) | 5  (12.50) | 40  (32.36) | 5  (5.68) | 88 (70.97) | 0  (0.00) | 0  (0.00) | 0  (0.00) | 0  (0.00) |
| **Milk and milk products** | 0  (0.00) | 6 (100.00) | 0  (0.00) | 15 (100.00) | 0  (0.00) | 0  (0.00) | 0  (0.00) | 0  (0.00) | 0  (0.00) | 2  (13.33) | 0  (0.00) | 14 (93.33) | 0  (0.00) | 0  (0.00) | 0  (0.00) | 0  (0.00) |
| **Eggs** | 11 (44.00) | 25 (100.00) | 237 (17.12) | 1384 (100.00) | 0  (0.00) | 0  (0.00) | 0  (0.00) | 0  (0.00) | 0  (0.00) | 0  (0.00) | 167 (97.09) | 172 (12.43) | 0  (0.00) | 0  (0.00) | 0  (0.00) | 0  (0.00) |
| **Meat and meat products** | 0  (0.00) | 10 (100.00) | 0  (0.00) | 436 (100.00) | 0  (0.00) | 0  (0.00) | 0  (0.00) | 0  (0.00) | 0  (0.00) | 0  (0.00) | 0  (0.00) | 0  (0.00) | 0  (0.00) | 0  (0.00) | 0  (0.00) | 0  (0.00) |
| **Fish and seafood** | 17 (23.29) | 73 (100.00) | 675 (17.11) | 3945 (100.00) | 0  (0.00) | 0  (0.00) | 0  (0.00) | 0  (0.00) | 0  (0.00) | 44  (1.12) | 514 (64.82) | 793 (20.10) | 0  (0.00) | 23  (0.58) | 0  (0.00) | 0  (0.00) |
| **Fats and oils** | 1  (5.56) | 18 (100.00) | 2  (0.71) | 281 (100.00) | 0  (0.00) | 0  (0.00) | 0  (0.00) | 0  (0.00) | 0  (0.00) | 187 (66.55) | 2  (12.50) | 16  (5.69) | 0  (0.00) | 0  (0.00) | 0  (0.00) | 1  (0.00) |
| **Sugar, syrups, and sweets** | 10 (55.56) | 18 (100.00) | 1164 (70.08) | 1661 (100.00) | 0  (0.00) | 0  (0.00) | 0  (0.00) | 0  (0.00) | 36 (94.74) | 38  (2.29) | 182 (26.88) | 677 (40.76) | 1  (10.00) | 10  (0.60) | 0  (0.00) | 0  (0.00) |
| **Soups, sauces, and seasonings** | 23 (88.46) | 26 (100.00) | 1649 (49.12) | 3357 (100.00) | 211  (100.00) | 211 (6.29) | 31  (0.00) | 31  (0.92) | 347 (100.00) | 347 (10.34) | 1287 (94.22) | 1366 (40.69) | 16 (100.00) | 16  (0.48) | 36 (100.00) | 36  (1.07) |
| **Beverages** | 24 (75.00) | 32 (100.00) | 346 (82.38) | 420 (100.00) | 0  (0.00) | 0  (0.00) | 20  (95.23) | 21  (5.00) | 235 (81.60) | 288 (68.57) | 90 (91.84) | 98 (23.33) | 0  (0.00) | 0  (0.00) | 45 (100.00) | 45 (10.71) |
| **Other** | 8  (42.11) | 19 (100.00) | 556 (90.85) | 612 (100.00) | 0  (0.00) | 31  (5.07) | 0  (0.00) | 48  (7.84) | 57  (55.34) | 103 (16.83) | 62 (87.32) | 71 (11.60) | 0  (0.00) | 0  (0.00) | 0  (0.00) | 3  (0.49) |
| **Total** | 170 (33.60) | 506 (100.00) | 7947 (29.51) | 26928 (100.00) | 211  (15.51) | 1360 (5.05) | 99  (8.12) | 1219 (4.53) | 2919 (55.75) | 5236 (19.44) | 4672 (56.66) | 8246 (30.62) | 18 (20.00) | 90  (0.33) | 82  (94.25) | 87  (0.32) |

Note: The percentage indicated in the UPF column indicates the share of UPF products consumed with missing values within each food group. The percentage indicated in the total column indicates the share of total products consumed that had missing values within each food group. No products had missing energy values.

**Appendix B.** Participants with a dietary intake of 0g for nutrients of concern, by high and low ultra-processed foods (UPF) consumption, and whether or not this was a true reflection of intake, or due to missing data on nutrient values in the food composition table

|  | Total number of participants (N=2521) with intake of 0g | | | Total number of participants included in regression analysis (n=2111) with intake being 0g | | | Number of participants included in regression analysis (n=2111) who actually consumed 0g | | | Number of participants included in regression analysis (n=2111) who have an intake set as 0g due to missing data | | |
| --- | --- | --- | --- | --- | --- | --- | --- | --- | --- | --- | --- | --- |
|  | **Low UPF consumer** | **High UPF consumer** | **Total** | **Low UPF consumer** | **High UPF consumer** | **Total** | **Low UPF consumer** | **High UPF consumer** | **Total** | **Low UPF consumer** | **High UPF consumer** | **Total** |
| Total fat | 2 | 0 | 2 | 1 | 0 | 1 | 1 | 0 | 1 | 0 | 0 | 0 |
| Saturated fat | 3 | 0 | 3 | 2 | 0 | 2 | 1 | 0 | 1 | 1 | 0 | 1 |
| Total sugar | 13 | 2 | 22 | 9 | 1 | 17 | 2 | 0 | 2 | 7 | 1 | 15 |
| Added sugar | 271 | 172 | 832 | 233 | 139 | 705 | 30 | 0 | 35 | 203 | 139 | 670 |
| Sodium | 2 | 0 | 2 | 1 | 0 | 1 | 0 | 0 | 0 | 1 | 0 | 1 |
| Fibre | 5 | 0 | 5 | 3 | 0 | 3 | 3 | 0 | 3 | 0 | 0 | 3 |

Note: This only table only reflects intake by nutrient intake was 0g. Participants who consumed more than 0g per day may still have some missing values (as reflected in Appendix A). High UPF consumers reflect those with the highest quartile of UPF consumption (for share of total energy), and low UPF consumers reflect those in the lowest quartile of UPF consumption. Totals do not necessarily equal the sum of low and high UPF consumers, as total number of participants also include moderate UPF consumers (quartile 2 and 3 of UPF intake).
